# Supplementary figures and images for: Super.Complex: A supervised machine learning pipeline for molecular complex detection in protein-interaction networks
Source: PLoS One. 2021 Dec 31;16(12):e0262056. doi: 10.1371/journal.pone.0262056 (PMC8719692; doi:10.1371/journal.pone.0262056)

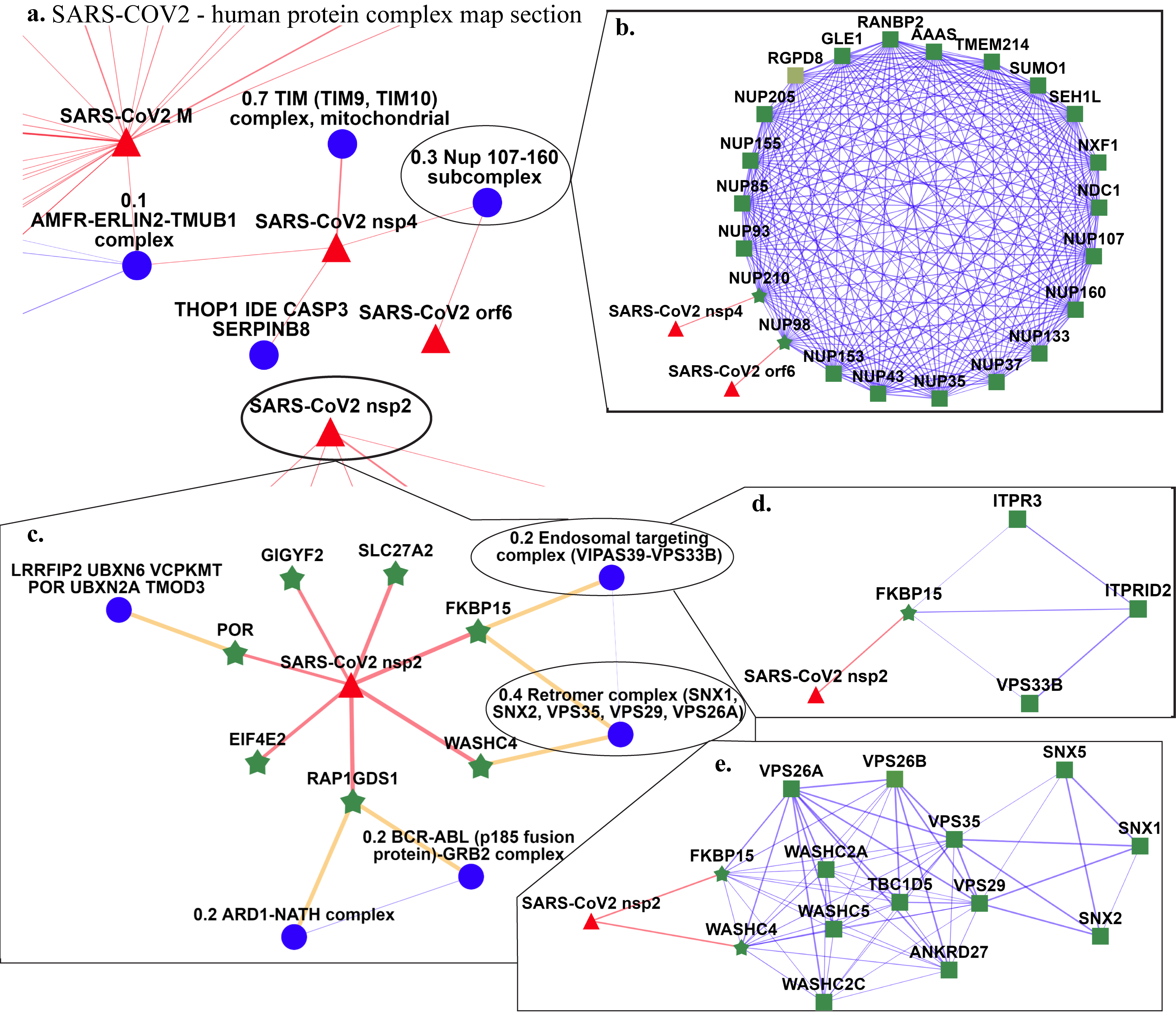

Supplement: S1 Fig — a. A section of the full map, featuring SARS-CoV-2 nsp4 and orf6 and their interacting human protein complexes b. A protein complex with a 30% match to the Nup 107–160 subcomplex interacts with both SARS-CoV-2 nsp4 and orf6 c. Map of SARS-CoV-2 nsp2 interactions with human proteins and their corresponding complexes d. A complex with a 20% match to the Endosomal targeting complex, and e. A complex with a 40% match to the retromer complex, both of which interact with SARS-CoV-2 nsp2. An interactive map is available at https://meghanapalukuri.github.io/Complexes/SARS_COV2_Map_only_mapped_complexes_names.html. (TIF) [file pone.0262056.s002.tif]
